# 8-Phenyl-3,4,6,7,8,8a-hexahydro-1*H*-pyrrolo­[2,1-*c*][1,4]oxazin-6-one

**DOI:** 10.1107/S1600536811032806

**Published:** 2011-08-27

**Authors:** Magdalena Małecka, Beata Pasternak, Stanisław Leśniak

**Affiliations:** a Department of Structural Chemistry and Crystallography, University of Łódź, Tamka 12, PL-91403 Łódź, Poland; b Department of Organic and Applied Chemistry, University of Łódź, Tamka 12, PL-91403 Łódź, Poland

## Abstract

In the title compound, C_13_H_15_NO_2_, the hexa­hydro­pyrrolo­[2,1-*c*][1,4]oxazine fragment is disordered over two conformations (*A* and *B*) in a 0.656 (5):0.344 (5) ratio. The five-membered ring is similarly disordered and adopts an envelope conformation in *A*, while in *B* this ring is nearly planar [maximum deviation = 0.088 (1) Å]. The six-membered rings in both *A* and *B* exhibit chair conformations. In the crystal, weak inter­molecular C—H⋯O hydrogen bonds link the mol­ecules into ribbons propagating in [010].

## Related literature

For the synthesis, see: Leśniak *et al.* (2009 ▶). For bond-length data, see: Allen *et al.* (1987 ▶). For the biological properties of similar structures, see: Nicolaou *et al.* (2002 ▶). For related structures, see: Chaume *et al.* (2008 ▶); Dorsey *et al.* (2003 ▶); Harwood *et al.* (1997 ▶).
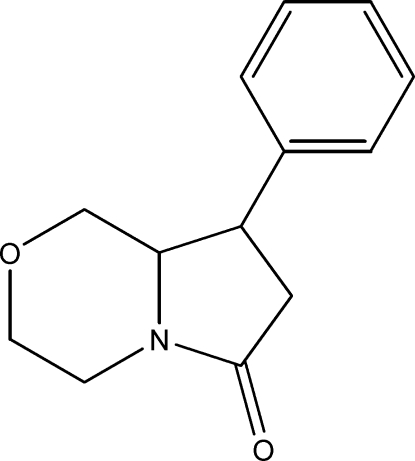


## Experimental

### 

#### Crystal data

C_13_H_15_NO_2_
*M* *_r_* = 217.27
Monoclinic, 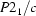

*a* = 13.2737 (12) Å
*b* = 7.1066 (4) Å
*c* = 11.9233 (10) Å
β = 103.917 (7)°
*V* = 1091.72 (15) Å^3^
*Z* = 4
Mo *K*α radiation
μ = 0.09 mm^−1^
*T* = 100 K
0.36 × 0.21 × 0.03 mm

#### Data collection

Stoe IPDS 2 diffractometer
6960 measured reflections
2301 independent reflections
1200 reflections with *I* > 2σ(*I*)
*R*_int_ = 0.108

#### Refinement

*R*[*F* ^2^ > 2σ(*F* ^2^)] = 0.047
*wR*(*F* ^2^) = 0.109
*S* = 0.81
2301 reflections
196 parameters
H atoms treated by a mixture of independent and constrained refinement
Δρ_max_ = 0.21 e Å^−3^
Δρ_min_ = −0.27 e Å^−3^

### 

Data collection: *X-AREA* (Stoe & Cie, 2000 ▶); cell refinement: *X-AREA*; data reduction: *X-RED* (Stoe & Cie, 2000 ▶); program(s) used to solve structure: *SHELXS97* (Sheldrick, 2008 ▶); program(s) used to refine structure: *SHELXL97* (Sheldrick, 2008 ▶); molecular graphics: *PLATON* (Spek, 2009 ▶); software used to prepare material for publication: *PLATON*.

## Supplementary Material

Crystal structure: contains datablock(s) I, global. DOI: 10.1107/S1600536811032806/cv5138sup1.cif

Structure factors: contains datablock(s) I. DOI: 10.1107/S1600536811032806/cv5138Isup2.hkl

Supplementary material file. DOI: 10.1107/S1600536811032806/cv5138Isup3.cml

Additional supplementary materials:  crystallographic information; 3D view; checkCIF report

## Figures and Tables

**Table 1:** Hydrogen-bond geometry (Å, °)

*D*—H⋯*A*	*D*—H	H⋯*A*	*D*⋯*A*	*D*—H⋯*A*
C2—H2*B*⋯O2^i^	0.97	2.46	3.329 (3)	149
C7*A*—H7*A*⋯O1^ii^	0.97	2.43	3.154 (4)	131

Symmetry codes: (i) 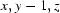; (ii) 
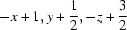
.

**Fig. 1.**
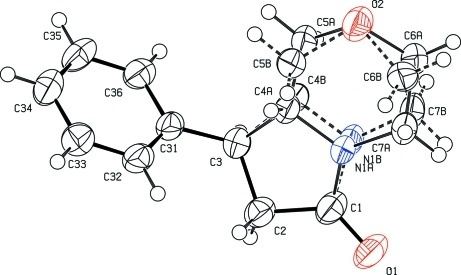
Molecular structure of I with the atom-numbering scheme. Displacement ellipsoids are drawn at the 50% probability level.

## supplementary crystallographic information

### Comment

In this paper we provide a new oxazin-6-on derivative prepared in one step synthesis in FVT (Leśniak *et al.*, 2009). The title compound (Fig. 1) represents an important structural unit found in biologically active compounds (Nicolaou *et al.*, 2002). The hexahydro-pyrrolo[2,1-*c*][1,4] oxazine fragment is disordered over two conformations - A and B, respectively - in a ratio 0.656 (5):0.344 (5). Disordered five-membered ring adopts an envelope conformation in A, while in B this ring is nearly planar. Six-membered ring in A and B exhibits a chair conformation. Bond lengths (Allen *et al.*, 1987) and angles are normal and correspond well to those observed in related structures (Chaume *et al.*, 2008; Dorsey *et al.*, 2003; Harwood *et al.*, 1997).

The packing of the molecules in the crystal lattice is stabilized *via* weak intermolecular C—H···O hydrogen bonds (Table 1), which link the molecules into ribbons propagated in [010].

### Experimental

General procedure. The flash vacuum thermolysis reactions were carried out in a 30x2.5 cm electrically heated horizontally oriented quartz tube packed with quartz rings, at 1.5x10^-3^ Torr. The synthetic precursor (*E*)-1-morpholin-4-yl-3-phenylprop-2-en-1-one (2 mmol) was slowly sublimed at 80–100°C from a flask held into thermolysis preheated to 950–1000°C. The product thereby obtain was collected in a CO_2_ acetone trap. After thermolysis, the whole system was brought to atmospheric pressure, allowing slow warming up to room temperature and the products were dissolved in CHCl_3_. The solvent was removed under reduced pressure and 8-phenyl-hexahydro- pyrrolo[2,1-*c*][1,4]oxazin-6-one was purified chromatographically on SiO_2_ and recrystallized from the hexane/CH_2_Cl_2_ (1:1) mixture.

### Refinement

The morpholin group was treated as disordered over two conformations with occupancies refined to 0.656 (5) and 0.344 (5), respectively. All H-atoms were positioned geometrically and refined with a riding model; for methine H atoms *U*_iso_ were constrained to be 1.2 times *U*_eq_ of the carrier atom and C—H=0.98 Å.

### Crystal data

**Table:**

C_13_H_15_NO_2_	*F*(000) = 464
*M**_r_* = 217.27	*D*_x_ = 1.322 Mg m^−^^3^
Monoclinic, *P*2_1_/*c*	Mo *K*α radiation, λ = 0.71073 Å
Hall symbol: -P 2ybc	Cell parameters from 2762 reflections
*a* = 13.2737 (12) Å	θ = 1.6–27.1°
*b* = 7.1066 (4) Å	µ = 0.09 mm^−^^1^
*c* = 11.9233 (10) Å	*T* = 100 K
β = 103.917 (7)°	Plate, colourless
*V* = 1091.72 (15) Å^3^	0.36 × 0.21 × 0.03 mm
*Z* = 4	

### Data collection

**Table:**

Stoe IPDS 2 diffractometer	1200 reflections with *I* > 2σ(*I*)
Radiation source: sealed X-ray tube, 12 x 0.4 mm long-fine focus	*R*_int_ = 0.108
planar graphite	θ_max_ = 26.8°, θ_min_ = 1.6°
Detector resolution: 6.67 pixels mm^-1^	*h* = −16→16
rotation method scans	*k* = −8→8
6960 measured reflections	*l* = −14→15
2301 independent reflections	

### Refinement

**Table:**

Refinement on *F*^2^	Secondary atom site location: difference Fourier map
Least-squares matrix: full	Hydrogen site location: inferred from neighbouring sites
*R*[*F*^2^ > 2σ(*F*^2^)] = 0.047	H atoms treated by a mixture of independent and constrained refinement
*wR*(*F*^2^) = 0.109	*w* = 1/[σ^2^(*F*_o_^2^) + (0.0509*P*)^2^] where *P* = (*F*_o_^2^ + 2*F*_c_^2^)/3
*S* = 0.81	(Δ/σ)_max_ < 0.001
2301 reflections	Δρ_max_ = 0.21 e Å^−^^3^
196 parameters	Δρ_min_ = −0.27 e Å^−^^3^
0 restraints	Extinction correction: *SHELXL*, Fc^*^=kFc[1+0.001xFc^2^λ^3^/sin(2θ)]^-1/4^
Primary atom site location: structure-invariant direct methods	Extinction coefficient: 0.047 (4)

### Special details

**Table:**

Geometry. All e.s.d.'s (except the e.s.d. in the dihedral angle between two l.s. planes) are estimated using the full covariance matrix. The cell e.s.d.'s are taken into account individually in the estimation of e.s.d.'s in distances, angles and torsion angles; correlations between e.s.d.'s in cell parameters are only used when they are defined by crystal symmetry. An approximate (isotropic) treatment of cell e.s.d.'s is used for estimating e.s.d.'s involving l.s. planes.
Refinement. Refinement of *F*^2^ against ALL reflections. The weighted *R*-factor *wR* and goodness of fit *S* are based on *F*^2^, conventional *R*-factors *R* are based on *F*, with *F* set to zero for negative *F*^2^. The threshold expression of *F*^2^ > σ(*F*^2^) is used only for calculating *R*-factors(gt) *etc*. and is not relevant to the choice of reflections for refinement. *R*-factors based on *F*^2^ are statistically about twice as large as those based on *F*, and *R*- factors based on ALL data will be even larger.

### Fractional atomic coordinates and isotropic or equivalent isotropic displacement parameters (Å^2^)

**Table:**

	*x*	*y*	*z*	*U*_iso_*/*U*_eq_	Occ. (<1)
C6A	0.6366 (3)	0.7343 (6)	0.8977 (3)	0.0425 (9)	0.656 (5)
H6A	0.6341	0.8221	0.8348	0.051*	0.656 (5)
H6B	0.5799	0.7624	0.9333	0.051*	0.656 (5)
C6B	0.6801 (6)	0.6628 (11)	0.8736 (5)	0.0464 (18)	0.344 (5)
H6C	0.7183	0.5561	0.8543	0.056*	0.344 (5)
H6D	0.6709	0.7541	0.8115	0.056*	0.344 (5)
C5A	0.7290 (3)	0.6480 (5)	1.0830 (3)	0.0409 (9)	0.656 (5)
H5A	0.6657	0.6814	1.1054	0.049*	0.656 (5)
H5B	0.7878	0.6762	1.1466	0.049*	0.656 (5)
C5B	0.7692 (5)	0.5623 (12)	1.0597 (6)	0.0437 (17)	0.344 (5)
H5C	0.7980	0.4684	1.0172	0.052*	0.344 (5)
H5D	0.8204	0.5922	1.1304	0.052*	0.344 (5)
C7A	0.6287 (3)	0.5338 (5)	0.8537 (3)	0.0410 (9)	0.656 (5)
H7A	0.5612	0.5128	0.8019	0.049*	0.656 (5)
H7B	0.6814	0.5110	0.8112	0.049*	0.656 (5)
C7B	0.5784 (6)	0.6015 (11)	0.8900 (6)	0.051 (2)	0.344 (5)
H7C	0.5428	0.7067	0.9152	0.061*	0.344 (5)
H7D	0.5356	0.5545	0.8178	0.061*	0.344 (5)
N1A	0.6434 (2)	0.4075 (4)	0.9511 (2)	0.0353 (7)	0.656 (5)
N1B	0.5961 (5)	0.4532 (9)	0.9771 (5)	0.0411 (15)	0.344 (5)
C4A	0.7283 (3)	0.4418 (5)	1.0511 (2)	0.0364 (9)	0.656 (5)
H4A	0.7948	0.4042	1.0360	0.044*	0.656 (5)
C4B	0.6714 (5)	0.4927 (9)	1.0858 (5)	0.0407 (18)	0.344 (5)
H4B	0.6440	0.5817	1.1339	0.049*	0.344 (5)
O1	0.52984 (10)	0.1832 (2)	0.86955 (11)	0.0594 (5)	
O2	0.73644 (10)	0.7476 (2)	0.98285 (11)	0.0568 (4)	
C36	0.81951 (14)	0.3636 (3)	1.33444 (16)	0.0453 (5)	
H36	0.7745	0.4576	1.3464	0.054*	
C2	0.64430 (14)	0.1486 (3)	1.06256 (15)	0.0448 (5)	
H2B	0.6954	0.0592	1.0491	0.054*	
H2A	0.5935	0.0820	1.0940	0.054*	
C32	0.86314 (15)	0.1182 (3)	1.22000 (16)	0.0459 (5)	
H32	0.8476	0.0445	1.1536	0.055*	
C35	0.91025 (14)	0.3310 (3)	1.41661 (16)	0.0514 (6)	
H35	0.9259	0.4034	1.4835	0.062*	
C33	0.95397 (15)	0.0859 (3)	1.30222 (18)	0.0516 (6)	
H33	0.9991	−0.0083	1.2908	0.062*	
C34	0.97791 (15)	0.1928 (3)	1.40096 (17)	0.0523 (6)	
H34	1.0392	0.1719	1.4566	0.063*	
C3	0.69621 (18)	0.3034 (3)	1.14461 (17)	0.0513 (6)	
C31	0.79475 (13)	0.2572 (3)	1.23386 (14)	0.0408 (5)	
C1	0.59269 (15)	0.2476 (3)	0.95258 (16)	0.0505 (5)	
H7	0.6478 (17)	0.348 (4)	1.1835 (19)	0.074 (8)*	

### Atomic displacement parameters (Å^2^)

**Table:**

	*U*^11^	*U*^22^	*U*^33^	*U*^12^	*U*^13^	*U*^23^
C6A	0.0416 (19)	0.041 (2)	0.0405 (18)	0.0053 (17)	0.0014 (14)	0.0030 (16)
C6B	0.060 (5)	0.038 (4)	0.039 (4)	0.002 (4)	0.008 (3)	−0.004 (3)
C5A	0.0446 (19)	0.043 (2)	0.0316 (16)	0.0039 (16)	0.0017 (14)	0.0012 (15)
C5B	0.040 (4)	0.040 (5)	0.046 (4)	−0.001 (3)	0.002 (3)	0.002 (3)
C7A	0.0405 (18)	0.047 (2)	0.0323 (17)	0.0032 (16)	0.0021 (13)	0.0030 (16)
C7B	0.052 (4)	0.061 (5)	0.033 (3)	0.003 (4)	−0.004 (3)	0.009 (3)
N1A	0.0368 (15)	0.0380 (16)	0.0278 (14)	0.0003 (12)	0.0012 (12)	0.0004 (11)
N1B	0.041 (3)	0.051 (4)	0.027 (3)	−0.006 (3)	0.000 (2)	0.004 (2)
C4A	0.0326 (17)	0.041 (2)	0.0320 (16)	0.0001 (15)	0.0012 (13)	−0.0001 (14)
C4B	0.039 (4)	0.046 (4)	0.034 (3)	−0.001 (3)	0.003 (3)	−0.003 (3)
O1	0.0560 (8)	0.0739 (11)	0.0405 (8)	−0.0204 (8)	−0.0034 (7)	−0.0059 (7)
O2	0.0596 (8)	0.0651 (10)	0.0396 (8)	−0.0191 (7)	0.0001 (6)	0.0087 (7)
C36	0.0479 (10)	0.0486 (12)	0.0393 (11)	−0.0088 (9)	0.0103 (8)	−0.0029 (9)
C2	0.0427 (10)	0.0501 (12)	0.0392 (11)	−0.0101 (9)	0.0052 (8)	−0.0007 (9)
C32	0.0566 (12)	0.0460 (13)	0.0338 (10)	−0.0097 (9)	0.0082 (9)	−0.0034 (9)
C35	0.0500 (12)	0.0702 (16)	0.0325 (11)	−0.0172 (11)	0.0070 (9)	−0.0101 (10)
C33	0.0476 (11)	0.0527 (14)	0.0522 (13)	−0.0031 (9)	0.0073 (10)	0.0005 (11)
C34	0.0449 (11)	0.0690 (16)	0.0389 (11)	−0.0107 (11)	0.0019 (9)	0.0050 (11)
C3	0.0674 (14)	0.0440 (13)	0.0347 (11)	−0.0032 (10)	−0.0031 (10)	0.0024 (10)
C31	0.0493 (10)	0.0421 (11)	0.0287 (10)	−0.0098 (9)	0.0050 (8)	0.0019 (9)
C1	0.0506 (11)	0.0627 (15)	0.0345 (11)	−0.0167 (10)	0.0027 (9)	0.0008 (10)

### Geometric parameters (Å, °)

**Table:**

C6A—O2	1.466 (3)	N1B—C4B	1.461 (7)
C6A—C7A	1.513 (6)	N1B—C1	1.489 (7)
C6A—H6A	0.9700	C4A—C3	1.618 (4)
C6A—H6B	0.9700	C4A—H4A	0.9800
C6B—O2	1.466 (7)	C4B—C3	1.516 (6)
C6B—C7B	1.476 (12)	C4B—H4B	0.9800
C6B—H6C	0.9700	O1—C1	1.220 (2)
C6B—H6D	0.9700	C36—C35	1.377 (2)
C5A—O2	1.411 (3)	C36—C31	1.389 (3)
C5A—C4A	1.513 (5)	C36—H36	0.9300
C5A—H5A	0.9700	C2—C1	1.500 (3)
C5A—H5B	0.9700	C2—C3	1.522 (3)
C5B—C4B	1.491 (10)	C2—H2B	0.9700
C5B—O2	1.603 (7)	C2—H2A	0.9700
C5B—H5C	0.9700	C32—C33	1.378 (3)
C5B—H5D	0.9700	C32—C31	1.378 (3)
C7A—N1A	1.444 (4)	C32—H32	0.9300
C7A—H7A	0.9700	C35—C34	1.373 (3)
C7A—H7B	0.9700	C35—H35	0.9300
C7B—N1B	1.459 (8)	C33—C34	1.373 (3)
C7B—H7C	0.9700	C33—H33	0.9300
C7B—H7D	0.9700	C34—H34	0.9300
N1A—C1	1.324 (3)	C3—C31	1.511 (3)
N1A—C4A	1.450 (4)	C3—H7	0.93 (2)
			
O2—C6A—C7A	106.0 (3)	C5B—C4B—C3	106.7 (6)
O2—C6A—H6A	110.5	N1B—C4B—H4B	111.9
C7A—C6A—H6A	110.5	C5B—C4B—H4B	111.9
O2—C6A—H6B	110.5	C3—C4B—H4B	111.9
C7A—C6A—H6B	110.5	C5A—O2—C6B	114.9 (3)
H6A—C6A—H6B	108.7	C5A—O2—C6A	108.5 (2)
O2—C6B—C7B	106.9 (6)	C6B—O2—C6A	34.4 (3)
O2—C6B—H6C	110.3	C5A—O2—C5B	33.9 (3)
C7B—C6B—H6C	110.3	C6B—O2—C5B	100.4 (4)
O2—C6B—H6D	110.3	C6A—O2—C5B	114.8 (3)
C7B—C6B—H6D	110.3	C35—C36—C31	120.5 (2)
H6C—C6B—H6D	108.6	C35—C36—H36	119.8
O2—C5A—C4A	105.7 (3)	C31—C36—H36	119.7
O2—C5A—H5A	110.6	C1—C2—C3	105.31 (17)
C4A—C5A—H5A	110.6	C1—C2—H2B	110.7
O2—C5A—H5B	110.6	C3—C2—H2B	110.7
C4A—C5A—H5B	110.6	C1—C2—H2A	110.7
H5A—C5A—H5B	108.7	C3—C2—H2A	110.7
C4B—C5B—O2	105.2 (5)	H2B—C2—H2A	108.8
C4B—C5B—H5C	110.7	C33—C32—C31	121.53 (18)
O2—C5B—H5C	110.7	C33—C32—H32	119.2
C4B—C5B—H5D	110.7	C31—C32—H32	119.2
O2—C5B—H5D	110.7	C34—C35—C36	120.81 (19)
H5C—C5B—H5D	108.8	C34—C35—H35	119.6
N1A—C7A—C6A	108.7 (3)	C36—C35—H35	119.6
N1A—C7A—H7A	109.9	C34—C33—C32	120.0 (2)
C6A—C7A—H7A	109.9	C34—C33—H33	120.0
N1A—C7A—H7B	109.9	C32—C33—H33	120.0
C6A—C7A—H7B	109.9	C33—C34—C35	119.22 (18)
H7A—C7A—H7B	108.3	C33—C34—H34	120.4
N1B—C7B—C6B	108.1 (5)	C35—C34—H34	120.4
N1B—C7B—H7C	110.1	C31—C3—C4B	125.0 (3)
C6B—C7B—H7C	110.1	C31—C3—C2	118.49 (18)
N1B—C7B—H7D	110.1	C4B—C3—C2	109.2 (2)
C6B—C7B—H7D	110.1	C31—C3—C4A	106.91 (19)
H7C—C7B—H7D	108.4	C4B—C3—C4A	37.5 (2)
C1—N1A—C7A	125.0 (2)	C2—C3—C4A	98.62 (18)
C1—N1A—C4A	115.5 (2)	C31—C3—H7	107.8 (14)
C7A—N1A—C4A	119.0 (3)	C4B—C3—H7	80.2 (16)
C7B—N1B—C4B	116.9 (5)	C2—C3—H7	107.7 (15)
C7B—N1B—C1	125.3 (5)	C4A—C3—H7	117.6 (16)
C4B—N1B—C1	110.2 (4)	C32—C31—C36	117.90 (17)
N1A—C4A—C5A	109.0 (2)	C32—C31—C3	123.72 (17)
N1A—C4A—C3	100.6 (2)	C36—C31—C3	118.36 (19)
C5A—C4A—C3	113.8 (3)	O1—C1—N1A	124.2 (2)
N1A—C4A—H4A	111.0	O1—C1—N1B	120.7 (3)
C5A—C4A—H4A	111.0	N1A—C1—N1B	33.7 (2)
C3—C4A—H4A	111.0	O1—C1—C2	127.8 (2)
N1B—C4B—C5B	108.8 (5)	N1A—C1—C2	106.68 (17)
N1B—C4B—C3	105.4 (4)	N1B—C1—C2	107.6 (2)

### Hydrogen-bond geometry (Å, °)

**Table:**

*D*—H···*A*	*D*—H	H···*A*	*D*···*A*	*D*—H···*A*
C2—H2B···O2^i^	0.97	2.46	3.329 (3)	149
C7A—H7A···O1^ii^	0.97	2.43	3.154 (4)	131

Symmetry codes: (i) *x*, *y*−1, *z*; (ii) −*x*+1, *y*+1/2, −*z*+3/2.
